# Opposite prognostic roles of HIF1α and HIF2α expressions in bone metastatic clear cell renal cell cancer

**DOI:** 10.18632/oncotarget.9669

**Published:** 2016-05-27

**Authors:** Attila Szendrői, A. Marcell Szász, Magdolna Kardos, Anna-Mária Tőkés, Roni Idan, Miklós Szűcs, Janina Kulka, Péter Nyirády, Miklós Szendrői, Zoltán Szállási, Balázs Győrffy, József Tímár

**Affiliations:** ^1^ Department of Urology, Semmelweis University, Budapest 1082, Hungary; ^2^ 2nd Department of Pathology, Semmelweis University, Budapest 1091, Hungary; ^3^ Molecular Oncology Research Group, Hungarian Academy of Sciences and Semmelweis University, Budapest 1091, Hungary; ^4^ Department of Orthopedics, Semmelweis University, Budapest 1113, Hungary; ^5^ Children's Hospital Informatics Program at the Harvard-MIT Division of Health Sciences and Technology, Harvard Medical School, Boston, MA 02115, USA; ^6^ Center for Biological Sequence Analysis, Department of Systems Biology, Technical University of Denmark, Lyngby 2800, Denmark; ^7^ MTA-TTK Lendület Cancer Biomarker Research Group, Budapest 1117, Hungary; ^8^ 2nd Department of Pediatrics, Semmelweis University, Budapest 1082, Hungary

**Keywords:** renal cell cancer, prognosis, bone metastasis, hypoxia inducible factor

## Abstract

**BACKGROUND:**

Prognostic markers of bone metastatic clear cell renal cell cancer (ccRCC) are poorly established. We tested prognostic value of HIF1α/HIF2α and their selected target genes in primary tumors and corresponding bone metastases.

**RESULTS:**

Expression of HIF2α was lower in mRCC both at mRNA and protein levels (p/mRNA/=0.011, p/protein/=0.001) while HIF1α was similar to nmRCC. At the protein level, CAIX, GAPDH and GLUT1 were increased in mRCC. In all primary RCCs, low HIF2α and high HIF1α as well as CAIX, GAPDH and GLUT1 expressions correlated with adverse prognosis, while VEGFR2 and EPOR gene expressions were associated with favorable prognosis. Multivariate analysis confirmed high HIF2α protein expression as an independent risk factor. Prognostic validation of HIFs, LDH, EPOR and VEGFR2 in RNA-Seq data confirmed higher HIF1α gene expression in primary RCC as an adverse (p=0.07), whereas higher HIF2α and VEGFR2 expressions as favorable prognostic factors. HIF1α/HIF2α-index (HIF-index) proved to be an independent prognostic factor in both the discovery and the TCGA cohort.

**PATIENTS AND METHODS:**

Expressions of HIF1α and HIF2α as well as their 7 target genes were analysed on the mRNA and protein level in 59 non-metastatic ccRCCs (nmRCC), 40 bone metastatic primary ccRCCs (mRCC) and 55 corresponding bone metastases. Results were validated in 399 ccRCCs from the TCGA project.

**CONCLUSIONS:**

We identified HIF2α protein as an independent marker of the metastatic potential of ccRCC, however, unlike HIF1α, increased HIF2α expression is a favorable prognostic factor. The HIF-index incorporated these two markers into a strong prognostic biomarker of ccRCC.

## INTRODUCTION

Among kidney cancers, clear cell renal cancer (ccRCC) is the histologically predominant form, which is a genetically heterogeneous malignancy [1]. A prominent pathological feature of ccRCC is its rich vasculature [2] due to the dysfunction of von Hippel-Lindau gene (VHL) and deregulation of *hypoxia inducible factors* (HIF) resulting in specific gene expression changes, which promote neoangiogenesis through *vascular endothelial growth factor* (VEGFs) and *vascular endothelial growth factor receptor* (VEGFRs) expressions [3]. Activity of HIFs further results in metabolic switch (affecting expression and function of *glucose transporter 1* (GLUT1), *glyceraldehyde-3-phosphate dehydrogenase* (GAPDH), *carbonic anhydrase 9* (CAIX), *erythropoietin receptor* (EPOR) and *lactate-dehydrogenase 5* (LDH5), providing a selection benefit for the tumor cells.

ccRCC is characterized by specific metastatic patterns, being lungs, liver and skeletal system the most frequently affected sites. Since metastatization is an organ selective process, it might rely on different geno- or phenotypes in various organs [4, 5]. In ccRCC, development of bone metastasis is considered a adverse prognostic factor [6]. A preliminary study raised the possibility that HIF1α and its target genes could be involved in shaping bone metastatic potential of ccRCC [7].

Although, the Fuhrman grading is still one of the best prognostic factors in ccRCC [8], this is also true in case of bone metastatic diseases [6]. Combined tools have been developed to predict therapy response, initially the Memorial Sloan-Kettering Cancer Center (MSKCC) nomogram stratified ccRCC into risk categories responsive to interferon therapy [9]. However, especially in the era of targeted treatment, the search for biomarkers continues, with several candidates emerging from the VHL-HIF pathway [10].

The simplistic VHL-HIF pathway driven angiogenic phenotype of ccRCC underwent a considerable redefinition recently. It was discovered by deep sequencing analysis that ccRCC can be further subclassified on the basis of mutations either of several transcriptional regulators, such as PBRM1 (*polybromo-1*), ARID1A (*AT-rich interactive domain-containing protein 1A*), BAP1 (*BRCA1 associated protein-1*), JARID2C (*lysine-specific demethylase 5C*), SETD2 (*SET domain containing 2*), or of the PTEN (*phosphatase and tensin homolog*)-mTOR (*mammalian target of rapamycin*) pathway members [11]. If transcriptional regulator mutations are also a hallmark of at least a large fraction of ccRCC we can postulate that the angiogenic phenotype of ccRCC may be defined at transcriptional levels as well, activating the expression of HIF family genes, which then control expression of the angiogenic genes. Since prognostic data are scanty on bone metastatic RCC, in our current study we analyzed mRNA- and protein expressions of HIF1α and HIF2α as well as their target genes in bone metastatic ccRCC to reveal their possible prognostic significance.

## RESULTS

### Clinicopathological characteristics

FFPE samples of fifty-five bone-metastatic (mRCC) and fifty-nine non-metastatic ccRCC (nmRCC) patients were investigated using their primary tumors and their respective bone metastases as a metastatic cohort (mRCC, Supplementary Table S1). The clinical data are presented on (Table 1).

**Table 1:** Clinicopathological characteristics of the patients with primary RCCs included in the study

*Variables/Group*		mRCC		nmRCC	
***Age (years)***	***Mean (range)***	61.08 (34-79)		60.87 (39-87)	
		n	%	n	%
***Gender***	***Male***	33	82	34	57
	***Female***	7	17	25	42
***Stage***	***1***	17	42	42	71
	***2***	7	17	9	15
	***3***	8	20	5	8
	***Unknown***	8	20	3	5
	***None***	0	0	59	100
***Metastases***	***Solitary osseal***	25	62	0	0
	***Multiplex osseal***	3	7	0	0
	***Osseal plus extra-osseal***	9	22	0	0
	***Unknown beyond osseal***	3	7	0	0
***Fuhrman Grade***	***1***	15	37	20	34
	***2***	16	40	31	52
	***3***	7	17	5	8
	***4***	2	5	3	5
***Overall survival (months)***	***Mean***	41.69		min.: 96.00 month	
	***Standard Deviation***	46.62		NA	

### Analysis of HIF1α, HIF2α and HIF-regulated gene expressions in primary ccRCCs

**Figure 1:**
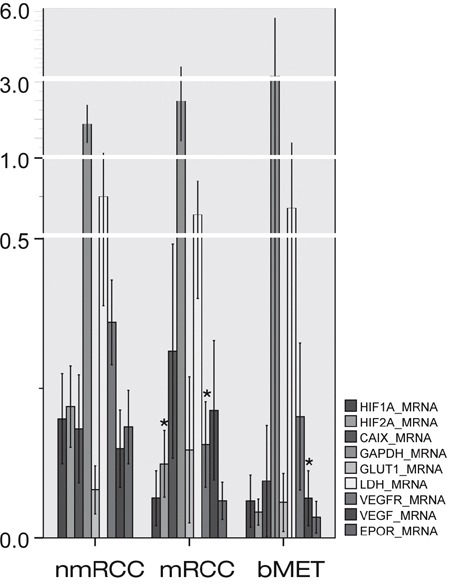
Expressions of HIF1α and HIF2α and their regulated genes at messenger ribonucleic acid (mRNA) level in primary non-metastatic (nmRCC) and metastatic renal cancer (mRCC) and in bone metastases (bMET) Asterisk means significant difference (metastatic vs. non-metastatic group, metastases vs. primary RCC, respectively); see p-values in text.

**Figure 2:**
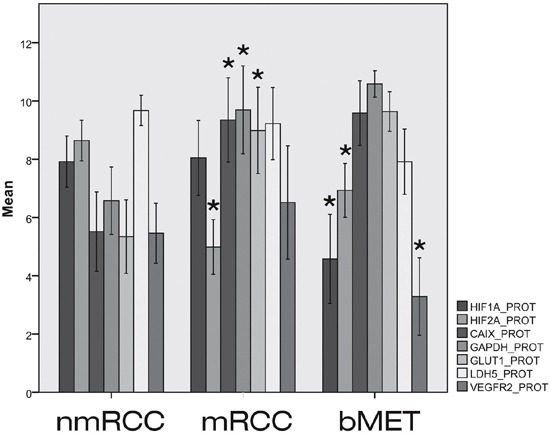
Expressions of HIF1α and HIF2α and their regulated genes at protein level in primary non-metastatic (nmRCC) and metastatic renal cancer (mRCC) and in bone metastases (bMET) Asterisk means significant difference (metastatic vs. non-metastatic group, metastases vs. primary RCC, respectively); see p-values in text.

While expression of HIF1α did not differ between the non-metastatic and bone-metastatic primary RCCs (p/mRNA/=0.252, p/protein/=0.385), HIF2α was found to be significantly lower both at mRNA and protein levels in the metastatic tumors (p/mRNA/=0.011, p/protein/=0.001) (Figures 1 and 2).

The mRNA expressions of CAIX, EPOR, GAPDH, GLUT1, LDH5 and VEGF did not differ significantly in the metastatic versus non-metastatic primary tumors (Figure 1). However, at protein levels CAIX (p=0.001), GAPDH (p=0.001) and GLUT1 (p=0.002) showed elevated expression in bone metastatic primary RCC. VEGFR2 data were controversial, showing decreased expression at gene expression level (p/mRNA/=0.001) in mRCC, which could not be confirmed by immunohistochemistry (p/protein/=0.294).

Correlation analysis of expression of HIFs and their regulated genes has been performed. At both mRNA (Pearson's correlation) and protein level (Spearman's rank correlation) statistical significance was reached for several markers in relation to HIF expression (Supplementary Figure 1). The investigated markers display a stronger correlation to HIF2α than to HIF1α (Table 2).

**Table 2:** Correlation of HIFs and their regulated genes and transcripts at mRNA (A) and protein (B) expression levels

A	mRNA	CAIX	EPOR	GAPDH	GLUT1	HIF1α	HIF2α	LDH	VEGFR	VEGF
**CAIX**	PC	1	0.26	0.244	0.828**	0.233	0.480**	0.256	0.255	0.452**
	p		0.057	0.06	0	0.078	0	0.05	0.057	0.001
**EPOR**	PC	0.26	1	0.986**	0.022	0.276*	0.595**	0.999**	0.227	0.251
	p	0.057		0	0.872	0.034	0	0	0.087	0.063
**GAPDH**	PC	0.244	0.986**	1	0.154	0.415**	−0.201	0.993**	0.279*	0.161
	p	0.06	0		0.243	0	0.124	0	0.026	0.203
**GLUT1**	PC	0.828**	0.022	0.154	1	0.135	0.431**	0.157	0.144	0.299*
	p	0	0.872	0.243		0.322	0.001	0.234	0.284	0.028
**HIF1α**	PC	0.233	0.276*	0.415**	0.135	1	0.543**	0.313*	0.182	−0.021
	p	0.078	0.034	0	0.322		0	0.011	0.154	0.869
**HIF2α**	PC	0.480**	0.595**	−0.201	0.431**	0.543**	1	0.02	0.667**	−0.006
	p	0	0	0.124	0.001	0		0.881	0	0.967
**LDH**	PC	0.256	0.999**	0.993**	0.157	0.313*	0.02	1	0.238	0.169
	p	0.05	0	0	0.234	0.011	0.881		0.058	0.185
**VEGFR**	PC	0.255	0.227	0.279*	0.144	0.182	0.667**	0.238	1	0.159
	p	0.057	0.087	0.026	0.284	0.154	0	0.058		0.221
**VEGF**	PC	0.452**	0.251	0.161	0.299*	−0.021	−0.006	0.169	0.159	1
	p	0.001	0.063	0.203	0.028	0.869	0.967	0.185	0.221	

B	Protein		HIF1α		CAIX	GAPDH	GLUT1	LDH5	VEGFR2	HIF2α
**HIF1α**	PC		1		−0.121	−0.023	−0.022	0.484**	0.420**	0.243**
	p		.		0.156	0.793	0.797	0	0	0.01
**CAIX**	PC		−0.121		1	0.655**	0.653**	0.007	−0.127	-0.213*
	p		0.156		.	0	0	0.941	0.154	0.027
**GAPDH**	PC		−0.023		0.655**	1	0.699**	−0.006	−0.08	-0.298**
	p		0.793		0	.	0	0.95	0.383	0.002
**GLUT1**	PC		−0.022		0.653**	0.699**	1	−0.082	0	-0.301**
	p		0.797		0	0	.	0.368	0.998	0.002
**LDH5**	PC		0.484**		0.007	−0.006	−0.082	1	0.388**	0.138
	p		0		0.941	0.95	0.368	.	0	0.157
**VEGFR2**	PC		0.420**		−0.127	−0.08	0	0.388**	1	0.109
	p		0		0.154	0.383	0.998	0	.	0.266
**HIF2α**	PC		0.243**		-0.213*	-0.298**	-0.301**	0.138	0.109	1
	p		0.01		0.027	0.002	0.002	0.157	0.266	.

** Correlation is significant at the 0.01 level (2-tailed).

* Correlation is significant at the 0.05 level (2-tailed).

p= Sig. (2 tailed).

PC= Correlation Coefficient.

### Survival analysis

**Figure 3:**
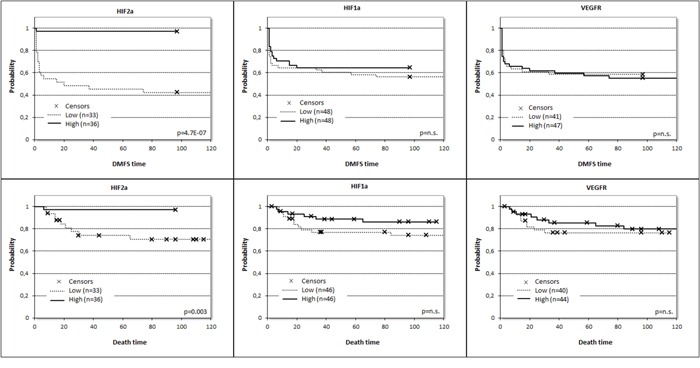
Prognostic potential for distant metastasis-free survival (DMFS) and overall survival (OS) related to expression of HIF1α (p_DMFS_= 0.435, HR_DMFS_ /±95%CI/ = 0.775 ± 0.639, p_OS_= 0.169, HR_OS_ /±95%CI/ = 0.497 ± 0.995), HIF2α (p_DMFS_= 0.001, HR_DMFS_/±95%CI/ = 0.035 ± 2.014, p_OS_= 0.019, HR_OS_/±95%CI/ = 0.085 ± 2.068) and VEGFR2 (p_DMFS_= 0.800, HR_DMFS_ /±95%CI/ = 1.085 ± 0.639, p_OS_= 0.609, HR_OS_ /±95%CI/ = 0.780 ± 0.952) at mRNA level split at the median values

A risk score was then generated for each marker based on model coefficients. Resultant predicted risk scores were not only dichotomized at median expression (Figure 3) but at the 25^th^ and 75^th^ percentile as well, and corresponding Kaplan-Meier survival curves were generated and compared by log-rank statistics (Supplementary Figure 2). Elevated HIF1α mRNA proved to be an adverse prognostic factor for both distant-metastasis-free survival (DMFS) and overall survival (OS), which was confirmed at protein level as well for DMSF. Furthermore, HIF2α levels were inversely correlated with prognosis both at mRNA (DMFS) and protein levels (DMFS and OS), but surprisingly low expression was correlated with adverse prognosis. The HIF-regulated genes displayed limited prognostic power split into groups at median expression level and 25^th^ and 75^th^ percentiles (Supplementary Figure 2). These genes were also tested when grouped into clusters based on a threshold at median expression level. Then, CAIX, GAPDH and GLUT1 were prognostic at protein level for DMFS (higher expression resulting in poorer prognosis). CAIX, EPOR, GLUT1, VEGFR2 and VEGF were prognostic at mRNA level for DMFS (Table 3). Interestingly, at mRNA level the higher expression of EPOR, GLUT1 and VEGFR2 predicted better survival (Table 3).

**Table 3:** Univariate analysis of both protein (A) and mRNA (B) expressions for predicting prognosis of distant metastasis-free (DMFS) and overall survival (OS)

A)				
DMFS (protein)	P	HR	95% CI for HR	
**CAIX**	**0.047**	1.895	1.01	3.558
**GAPDH**	**0.002**	2.735	1.44	5.194
**GLUT1**	**0.013**	2.259	1.19	4.286
**HIF1α**	**0.005**	1.481	1.428	1.537
**HIF2α**	**<0.001**	0.083	**0.026**	0.272
**LDH5**	0.811	0.926	0.493	1.739
**VEGFR2**	0.994	1.002	0.521	1.929

OS (protein)	P	HR	95% CI for HR	
**CAIX**	0.132	2.08	0.802	5.395
**GAPDH**	0.066	2.45	0.944	6.358
**GLUT1**	0.202	1.861	0.717	4.828
**HIF1α**	**0.026**	1.241	1.028	1.402
**HIF2α**	**0.007**	0.131	**0.03**	0.574
**LDH5**	0.753	0.858	0.331	2.225
**VEGFR2**	0.427	0.679	0.262	1.762

B)				
DMFS	P	HR	95% CI for HR	
**CAIX**	**0.004**	2.69	1.36	5.323
**EPOR**	**0.001**	0.286	0.139	0.59
**GAPDH**	0.582	0.838	0.446	1.573
**GLUT1**	**0.015**	0.438	0.224	0.853
**HIF1α**	**0.023**	2.03	1.401	2.618
**HIF2α**	**<0.001**	0.109	**0.042**	0.28
**LDH**	0.219	0.67	0.354	1.269
**VEGF**	**0.007**	2.559	1.294	5.061
**VEGFR**	**<0.001**	**0.036**	**0.009**	0.149

OS	P	HR	95% CI for HR	
**CAIX**	0.548	1.156	0.72	1.858
**EPOR**	0.655	0.898	0.559	1.442
**GAPDH**	0.759	1.076	0.675	1.715
**GLUT1**	0.81	0.944	0.59	1.51
**HIF1α**	0.647	0.895	0.557	1.438
**HIF2α**	0.434	0.823	0.505	1.341
**LDH**	0.762	1.075	0.673	1.716
**VEGF**	0.349	1.252	0.783	2.001
**VEGFR**	0.581	0.863	0.512	1.456

The groups were split at median expression level into “high and low” expressing clusters.

To further test the power of HIF2α mRNA and protein expressions as prognostic factor in bone metastatic ccRCC, multivariate analysis was performed taking into account the available independent clinicopathological variables (gender, Fuhrman grade). According to this analysis, decreased HIF2α protein expression level remained an independent negative prognostic factor and outperformed all other clinicopathological factors, even HIF1α in multiple models (Table 4).

**Table 4:** Multivariate analysis of the prognostic role of hypoxia inducible factor (HIF) protein expressions in primary metastatic and non-metastatic clear cell renal cell cancer (ccRCC) in two comparisons

	p	HR	95% CI for HR	
			Lower	Upper
***HIF2α***	***0.042***	***0.11***	***0.01***	***0.92***
*Gender*	0.726	0.74	0.14	3.79
*Fuhrman Grade*	0.896	1.05	0.49	2.21
	**p**	**HR**	**95% CI for HR**	
			**Lower**	**Upper**
***HIF2α***	***0.036***	***0.10***	***0.01***	***0.86***
*HIF1α*	0.164	0.35	0.08	1.52
*Fuhrman Grade*	0.965	1.01	0.49	2.09

HR: hazard ratio, 95%CI: 95% confidence interval. HIF1α and HIF2α: hypoxia inducible factor 1α and 2α.

**Figure 4:**
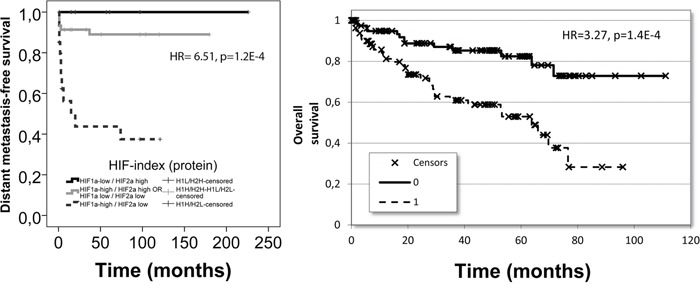
The prognostic performance of the identified HIF-index constructed from HIF1α and HIF2α: in our patient cohort for distant metastasis-free survival at protein level (left) and in the TCGA dataset for overall survival at mRNA level (right)

We also tested the combined prognostic power of HIF1α/HIF2α protein expressions. Survival analysis indicated that RCC patients characterized by HIF1α-high/HIF2α-low marker profile had the worst outcome while the opposite was found for patients belonging to the HIF1α-low/HIF2α-high group (Figure 4). The HIF1α-high/HIF2α-low or the HIF1α-low/HIF2α-low groups were found to fall in between the two previous prognostic groups (Table 5).

**Table 5:** Multivariate analysis of the prognostic role of HIF-index at protein expression level in the currently utilized FFPE samples and the TCGA cohort

*FFPE cohort*	p	HR	95% CI for HR	
			Lower	Upper
***HIF-index***	**0.022**	**3.576**	**1.203**	**10.624**
*Gender*	0.280	0.534	0.171	1.667
*Stage*	0.379	1.319	0.711	2.448
*Fuhrman Grade*	0.435	0.773	0.405	1.476
***TCGA cohort***	**p**	**HR**		
***HIF-index***	**0.048**	**2.05**		
***Age***	**0.016**	**1.03**		
*Gender*	0.43	0.74		
***Fuhrman Grade***	**0.032**	**1.72**		
***Stage***	**<0.001**	**2.05**		

HR: hazard ratio, 95%CI: 95% confidence interval. HIF-index: HIF1α and HIF2α index.

### Validation cohort

**Figure 5:**
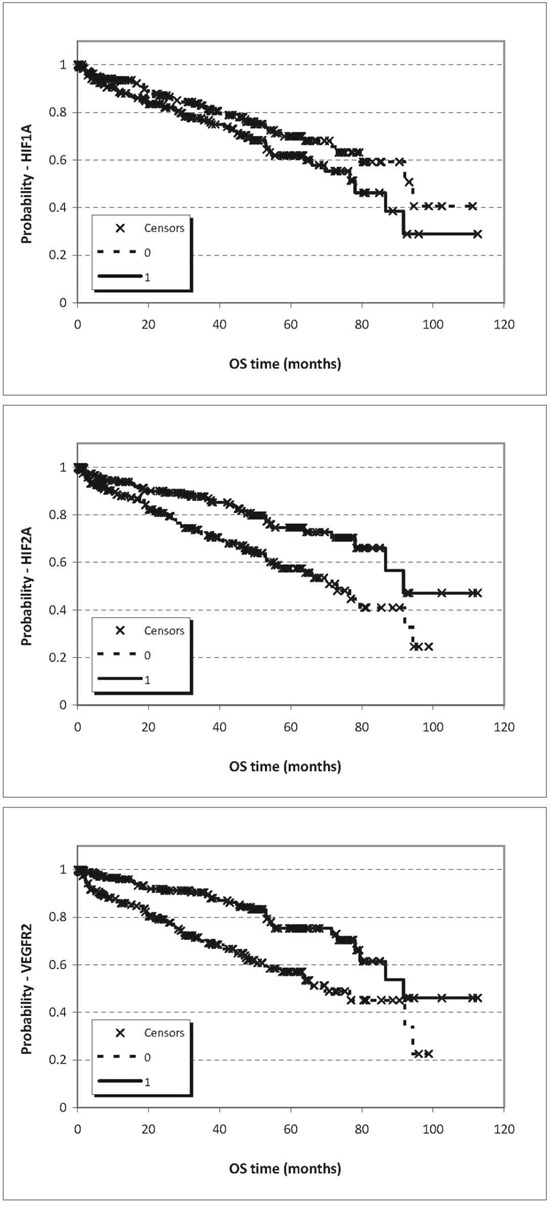
Prognostic potential for OS related to expression of HIF1α (p=0.07), HIF2α (p=8.1E-04), VEGFR2 (p=6.57E-05) at mRNA level based on Chip-seq data split at the median values

We tested the prognostic power of mRNA expression of HIF1α, HIF2α and VEGFR2 in the publicly available RNA-Seq data of ccRCC from the TCGA project involving 399 patients (Figure 5). In this cohort the distribution of available clinicopathological characteristics was similar to our cohort (p/gender/=1.000), however, in the TCGA cohort bone metastatic cases could not be filtered since the localization of metastases was not disclosed and therefore contained cases not limited to skeletal metastases. Survival analysis for HIF1α expression confirmed that higher expression (split at median) predicted poor survival (p=0.07, HR=1.41), while the opposite was found for HIF2α: increased expression was associated with improved overall survival (p<0.001, HR=0.506) and VEGFR2 expression behaved similarly to HIF2α in this cohort: high expression (<median) predicted favorable prognosis (p<0.001, HR=0.436). In this cohort again, the combination of HIF1α and HIF2α expressions provided the best prognostic power: the HIF1α-high/HIF2α-low patient group showed significantly poorer survival as compared with HIF1α-low/HIF2α-high patients (p<001, HR=3.27) (Table 5, Figure 4).

### Analysis of HIF1α and HIF2α and HIF-regulated genes in bone metastases compared with primary RCC

Neither HIF1α nor HIF2α mRNA expressions were found to be altered in bone metastases (Figure 1). However, HIF1α protein was shown to be significantly decreased (p/protein/=0.001) while HIF2α protein as determined by IHC was increased as compared with the primary site (p/protein/=0.023). None of the HIF-regulated genes or their protein products showed altered expressions in bone metastases (Figure 2), except for decreased VEGFR2 protein (p/protein/=0.006), and VEGF mRNA levels (p/mRNA/=0.020). The expressions of HIF1α and HIF2α and their regulated genes were also evaluated in relation to overall survival, as estimated from the time of operation of the bone metastasis. None of the investigated markers showed predictive power when split at median or mean values, either at mRNA or protein level (data not shown).

## DISCUSSION

Despite the growing knowledge on the association between HIF1α and HIF2α dysfunction and ccRCC, limited literature is available on the role of HIF(s) as prognostic or predictive markers. In most studies, HIF1α was analyzed at the protein level using immunohistochemistry and nuclear overexpression was found to be associated with adverse prognosis [10, 16–19]. Other studies identified cytoplasmic HIF1α or HIF2α [20] as a prognostic marker while in localized ccRCC, HIF1α did not prove to be part of the prognostic signature [10]. In a recent metaanalysis of 1258 RCC patients on the prognostic significance of HIF1α or HIF2α found high nuclear HIF1α expression was associated with poor overall survival, while high cytoplasmic expression of HIF2α was associated with poor cancer specific survival [19]. A previous study on 168 ccRCCs observed overexpression of HIF2α mRNA, which was associated with T stage and nuclear grade but not found to improve prognostication [21].

There is increasing controversy over the prognostic significance of HIF-regulated genes with the exception of CAIX, the protein expression of which is the most reliable marker of favorable prognosis [22, 23]. Several studies found VEGF-A overexpression as adverse prognostic marker [17, 24, 25], while others opted for VEGFR1 [17]. In summary, data are either controversial or missing on various HIFs and on HIF-regulated gene expressions as prognostic markers in ccRCC, especially regarding bone metastatic diseases.

In the current study, we provided evidences that the prognostic roles of HIF1α and HIF2α are different in bone metastatic RCC: increased HIF1α and decreased HIF2α have adverse prognostic potential. However, only decreased HIF2α expression proved to be an independent prognostic marker. Since the validation cohort (TCGA) contained various visceral metastases and our results based on a bone metastatic cohort, we can assume that our results could be generalized to all metastatic RCCs. A novel finding of our study is that we provided evidence that protein and mRNA expressions of HIF1α are not altered in metastatic and non-metastatic primary ccRCC. However, its prognostic potential is clear, suggesting that beside VHL mutations other disturbances of the regulation of gene expressions - most probably mutations of transcription regulatory genes (PBRM1, ARID1A, BAP1, JARID2C, and SETD2) - affect their expression as well. [26] A further possible cause of the inverse dysregulation of HIF-1α and HIF-2α might be the defects of the genes (SETD2, JARID1C (*lysine-specific demethylase 5C*), UTX (*ubiquitously transcribed tetratricopeptide repeat, X chromosome*), etc.) of histone modification, which are known to be common in wild-type VHL RCC. They observed opposite functional roles for HIF-1α and HIF-2α. HIF-1α regulated genes were associated with favorable prognosis while HIF-2α-regulated genes were associated with adverse prognosis. These data shows the existence of HIF-isoform-specific binding preferences of the regulated genes [27].

In accordance, our data confirmed the controversial roles of HIF-regulated genes as prognostic marker, since two out of seven HIF-regulated genes (CAIX, GLUT1) were found to be increased at mRNA and protein levels in bone metastatic primary ccRCC, and also had prognostic relevance, even though at protein and mRNA data seem contradictory, which needs further clarification. In an independent TCGA dataset of visceral metastatic RCCs, we have found that elevated VEGFR2 expression is a marker of favorable prognosis, which was confirmed in our bone metastatic cohort.

Bone metastasis of ccRCC is a frequent complication but relatively few studies have attempted to identify specific prognostic markers for this disease. Preliminary microarray data suggested HIF1α overexpression in parallel with its target genes VEGFR1, VEGFR2 to be involved [7]. Since we had access to bone metastases of ccRCCs, we ran this analysis on metastatic tissues as well, with the finding of decreased VEGFR2 and VEGF expressions. These data may suggest that bone metastases of ccRCCs may not be the best targets for antiangiogenic agents and provide a possible explanation for the relative therapy resistance.

Our study seems to be the first to clearly identify HIF2α as a significant marker of the (bone) metastatic potential of ccRCC. Several studies have recently found that HIF1α and HIF2α may be key regulators of the malignant phenotype of ccRCC with the finding of profound differences in their functions [26–28]. We also demonstrated that the combination of HIF1α and HIF2α expressions (either at mRNA or protein levels) provides the best prognostic power in RCCs. Today the dichotomization of ccRCCs into favorable and adverse prognostic groups is achieved by using MSKCC nomogram or a newly developed gene signature [29]. The question still remains whether the angiogenic phenotype of ccRCC is linked to prognosis. Our data on VEGFR2 expression linked to HIF2α rather than HIF1α expression suggest that the angiogenic phenotype of ccRCC may define the favorable prognosis subgroup, which can be more sensitive to antiangiogenic agents while the HIF1α-high/HIF2α-low subgroup may define the adverse prognosis subgroup of ccRCC, where VEGFR2 expression is also low. Whether this later subgroup can be linked to AKT (*RAC-gamma serine/threonine-protein kinase*) pathway alterations could well be the topic of further studies.

### Conclusion

In our study, we identified HIF2α as an independent marker of the metastatic potential of ccRCC, however, unlike HIF1α, increased HIF2α expression is a favorable prognostic factor. The HIF-index is incorporating these two markers into a strong predictive biomarker validated in both cohorts.

## MATERIALS AND METHODS

### Patients and samples

We collected samples from 55 metastatic clear cell renal cell carcinoma (ccRCC) patients who have been operated at the Department of Orthopedics between 1990-2008 for bone metastases. The primary tumors were gathered from pathological departments from all over the country for 40 cases (Table 1). Additionally, 59 ccRCC patients with no relapse on 102 months follow-up were selected from the archives of the Department of Urology and 2nd Department of Pathology as control group (Table 1). Laboratory data for serum lactate dehydrogenase (LDH) was not available for all the patients, therefore the patients could not be classified according to the MSKCC criteria.

Our study is retrospective in design, and represents patients from the pre-antiangiogenic drug-era. The patients with metastatic tumors were postoperatively treated with clodronate 2×800mg per os/day and 9M IU interferon alpha (IFNα) subcutaneously and vinblastine according to the applying guidelines [12]. In that period, the targeted therapies were not available in our country. The institutional review board (IKEB #185/2007) approved the study. Signed informed consents of the patients are not needed since the retrospective design of the study and the anonym data management.

The investigated tissues were formalin-fixed, paraffin-embedded (FFPE) material reviewed independently by three investigators (A.M.S., M.K., J.T.). TNM (Tumor Node Metastasis) classification was updated according to the 2009 TNM system and the grade was defined according to Fuhrman by a single renal pathologist (M.K.) [8]. After evaluation of cases for cellularity, 5 to 20 pieces of 5 μm thick sections were cut from the blocks for nucleic acid isolation. In all cases where the tumor tissue was available after sectioning, 2 cores with 2 mm diameter were selected and punched for tissue microarray (TMA) construction (TMA Master, 3DHistech Ltd., Budapest, Hungary).

### RNA purification and real-time polymerase chain reaction (qPCR)

RNA was extracted with QiagenRNeasy FFPE kit according to the manufacturer's protocol. The amount of RNA was measured with ND-1000 Spectrophotometer (NanoDrop Products, Wilmington, DE, USA). High Capacity RNA-to-cDNA Master Mix from Applied Biosystems (ABI, Foster City, CA, USA) was used for transcription of 1000 ng RNA in 20 μl final volume for each case. The epMotion 5070 pipetting system (Eppendorf, Hamburg, Germany) was used to transfer ingredients of the PCR reaction to full-skirted white 384-well plates from Roche (Roche Diagnostics, Basel, Switzerland). The final volume was 20 μl, containing the sample (2 μl), water (7 μl), TaqMan GeneExpression Master Mix (10 μl) and the adequate primers and hydrolysis probes (1 μl) for HIF2α; GAPDH, GLUT1; LDH5; EPOR; CAIX; VEGFR2; Beta-2-microglobulin (B2M); HIF1α; VEGF (all from ABI, Taqman Gene Expression Assay IDs, respectively: Hs01026142_m1; Hs02758991_g1; Hs00892681_m1; Hs00855332_g1; Hs00959432_g1; Hs00154208_m1; Hs00911705_g1; Hs02758991_g1; Hs00984230_m1; Hs00936371_m1; Hs00173626_ml). The reactions were running in a Lightcycler 480 real-time PCR system (Roche). The PCR program ran as follows: 10 min at 95°C for enzyme activation and DNA denaturation, and 40 PCR quantification cycles consisting of 95°C for 15 s and 60°C for 60 s. The raw data were exported in portable document format and converted to an Excel file (Microsoft Corp., Redmond, WA, USA). B2M (*beta-2-microglobulin*) was used as reference gene for the qPCR reactions [13].

### Immunohistochemistry (IHC)

Reactions were performed on 4 μm thick sections after deparaffination and antigen retrieval for 30 minutes in Target Retrieval Solution (DAKO, Carpinteria, CA, USA). Visualization of HIF2α (NB100-132H, Novus Biologicals, Littleton, CO, USA), HIF1α, CAIX, GAPDH, GLUT1, LDH5 and VEGFR2 (ab85886, ab15086, ab9484, ab652, ab8365, ab9484 and ab39638, respectively, all from AbCam, Cambridge, UK) reactions was performed using Novolink Detection System (Leica Microsystems, Wetzlar, Germany) or Ventana Benchmark automated immunostainer (in case of CAIX and GLUT1). VEGF and EPOR antibodies were not available in a validated manner on the market, thus, were not utilized. Slides were digitalized with Mirax MIDI scanner and evaluated with TMA modul software in MiraxViewer (v1.11, 3DHistech). Frequency of the reaction was scored on a 0-8 scale, intensity on a 0-3 scale measure. For further calculations, these two scores were summarized resulting in a 0-11 combined score system.

### Independent dataset for validation and computation

To assess the above genes' expression in a separate patient cohort, we evaluated publicly available microarray profiles. For identifying relevant gene chips we searched the GEO (http://www.ncbi.nlm.nih.gov/geo) and The Cancer Genome Atlas (TCGA) (http://cancergenome.nih.gov/) repositories using the keywords “RCC”, “renal cell cancer” and “gpl96”, “gpl570” and “gpl571”. With these criteria, no datasets were published in the GEO or TCGA repository. We therefore used the RNA-Seq data for renal cancer patients published in the TCGA project (http://cancergenome.nih.gov/) and downloaded the pre-processed level 3 data generated using Illumina HiSeq 2000 RNA Sequencing Version 2 platform. In these samples, gene expression levels were computed using a combination of MapSplice and RSEM (RNA-Seq by Expectation-Maximization). We combined individual patient files in R using the plyr package [14]. The following RNA-Seq probes (Symbol/Illumina RNA-Seq ID) were used in the analysis: HIF1α/3091, HIF2α/2034, LDHA/3939, EPOR/2057 and VEGFR2/3791.

The complete published RCC database contained 502 patients. The 86 patients who received neoadjuvant therapy (n=18), immunotherapy (n=28), tyrosine kinase inhibitor therapy (n=30) or chemotherapy (n=10) were excluded from the final analysis. Overall survival data was available for calculation for 399 patients. Follow-up period in case of the remaining patients was 36.6 ± 26.9 months, with age 61.0 ± 12.4 months and 66% being male and 34% female.

### Statistics

The qPCR and IHC data analysis was performed with SPSS 15.0 (SPSS Inc., Chicago, IL, USA). The “2^(ΔΔCq)” method and student's T-test was applied for comparing relative expression results (qPCR values) between the investigated groups [15]. For non-parametric values (immunohistochemistry) Mann-Whitney U-test was used. Kaplan-Meier graphs were plotted to visualize the results using expression values as a cut-off determining high and low expression cohorts. Cox proportional hazard regression was performed in the tumor samples to compare the association between mRNA/protein expression and distant metastasis-free (DMFS) and overall survival (OS), using WinSTAT 2007 for Microsoft Excel (Robert K. Fitch Software, Germany) for datasets and SPSS 15.0 for clinical sample data. The ‘p' values less than 0.05 were considered as being statistically significant.

## SUPPLEMENTARY FIGURES AND TABLES


