# Supplementary material for: Opposite prognostic roles of HIF1α and HIF2α expressions in bone metastatic clear cell renal cell cancer
Source: Oncotarget. 2016 May 27;7(27):42086–98. doi: 10.18632/oncotarget.9669 (PMC5173118; doi:10.18632/oncotarget.9669)
Supplement: Supplementary file 1 [file oncotarget-07-42086-s001.pdf]

SUPPLEMENTARY FIGURES AND TABLE

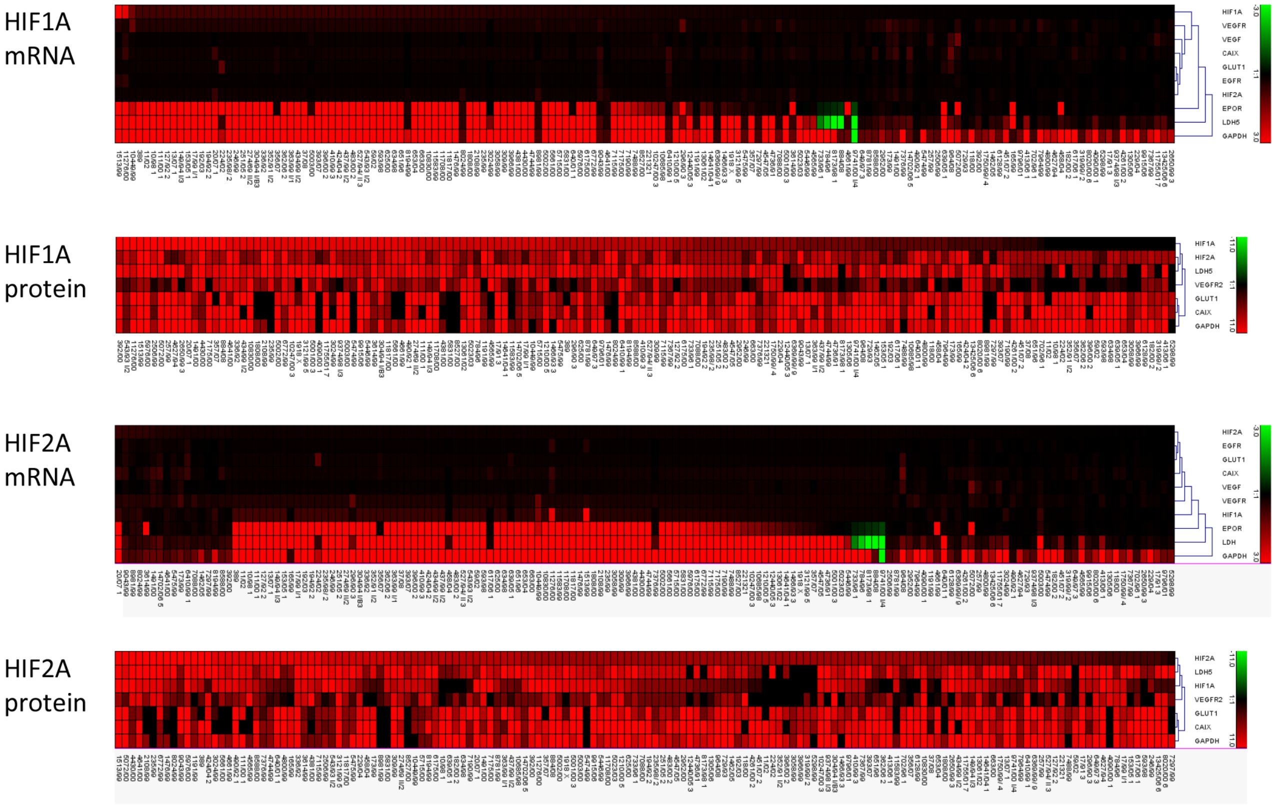

Supplementary Figure S1: Heatmap representation of expression of HIF1α and HIF2α both at mRNA and protein level and all the genes and proteins supposedly regulated by HIFs. Correlation analysis was performed which supports the regulation of the selected markers by HIFs.

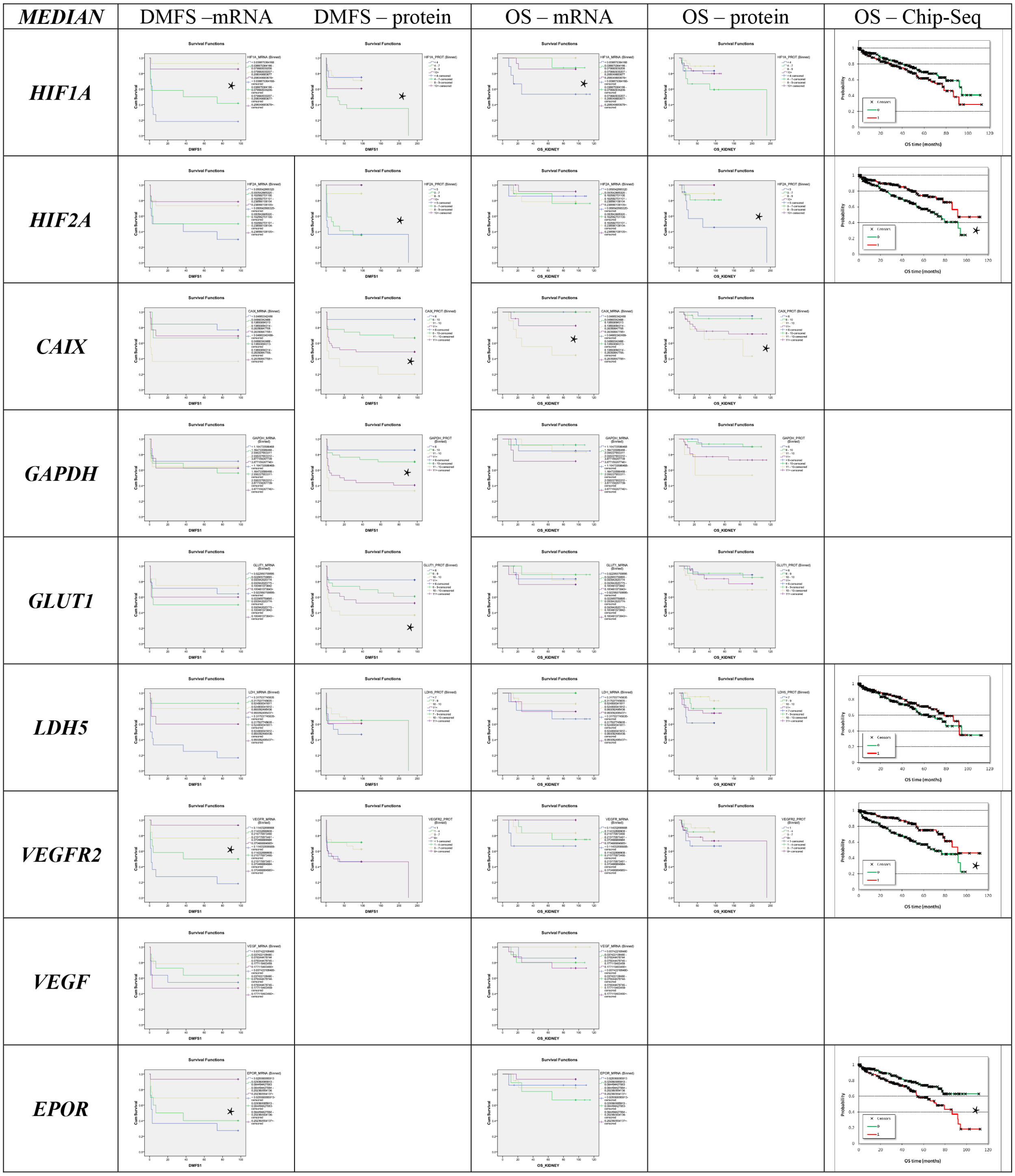

Supplementary Figure S2: Kaplan-Meier estimates of the evaluated factors. Resultant predicted risk scores were not only dichotomized at median expression but at the 25th and 75th percentile as well. Asterisk means significant difference in survival comparing the above expression level based groups.

Supplementary Table S1: Abbreviations

HIF1α	hypoxia inducible factor 1 alpha
HIF2α	hypoxia inducible factor 2 alpha
ccRCC	clear cell renal cell cancer
mRNA	messenger ribonucleic acid
mRCC	metastatic primary clear cell renal cell cancers
nmRCC	non metastatic primary clear cell renal cell cancers
GLUT1	glucose transporter 1
CAIX	carbonic anhydrase 9
GAPDH	glyceraldehyde-3-phosphate dehydrogenase
LDH5	lactate-dehydrogenase 5
EPOR	erythropoietin receptor
VEGFR2	vascular endothelial growth factor receptor 2
VEGF	vascular endothelial growth factor
TCGA	The Cancer Genome Atlas
DMFS	distant metastasis-free survival
VHL	von Hippel-Lindau
MSKCC	Memorial Sloan-Kettering Cancer Center
PBRM1	polybromo-1
ARID1A	AT-rich interactive domain-containing protein 1A
BAP1	BRCA1 associated protein-1
SETD2	SET domain containing 2
PTEN	phosphatase and tensin homolog
mTOR	mammalian target of rapamycin
IFNα	interferon alpha
IKEB	institutional review board
FFPE	formalin-fixed, paraffin-embedded
TNM	Tumor Nodes Metastasis
TMA	tissue microarray
qPCR	real-time polymerase chain reaction
cDNA	complementary DNA
B2M	Beta-2-microglobulin
IHC	Immunohistochemistry
RSEM	RNA-Seq by Expectation-Maximization
OS	overall survival
JARID2C	lysine-specific demethylase
UTX	ubiquitously transcribed tetratricopeptide repeat, X chromosome
AKT	RAC-gamma serine/threonine-protein kinase
bMET	bone metastases
